# Supplementary material for: The canonical E2Fs together with RETINOBLASTOMA-RELATED are required to establish quiescence during plant development
Source: Commun Biol. 2023 Sep 4;6:903. doi: 10.1038/s42003-023-05259-2 (PMC10477330; doi:10.1038/s42003-023-05259-2)
Supplement: Supplementary file 2 — Supplementary Information File [file 42003_2023_5259_MOESM2_ESM.pdf]

Supplementary Fig. 1

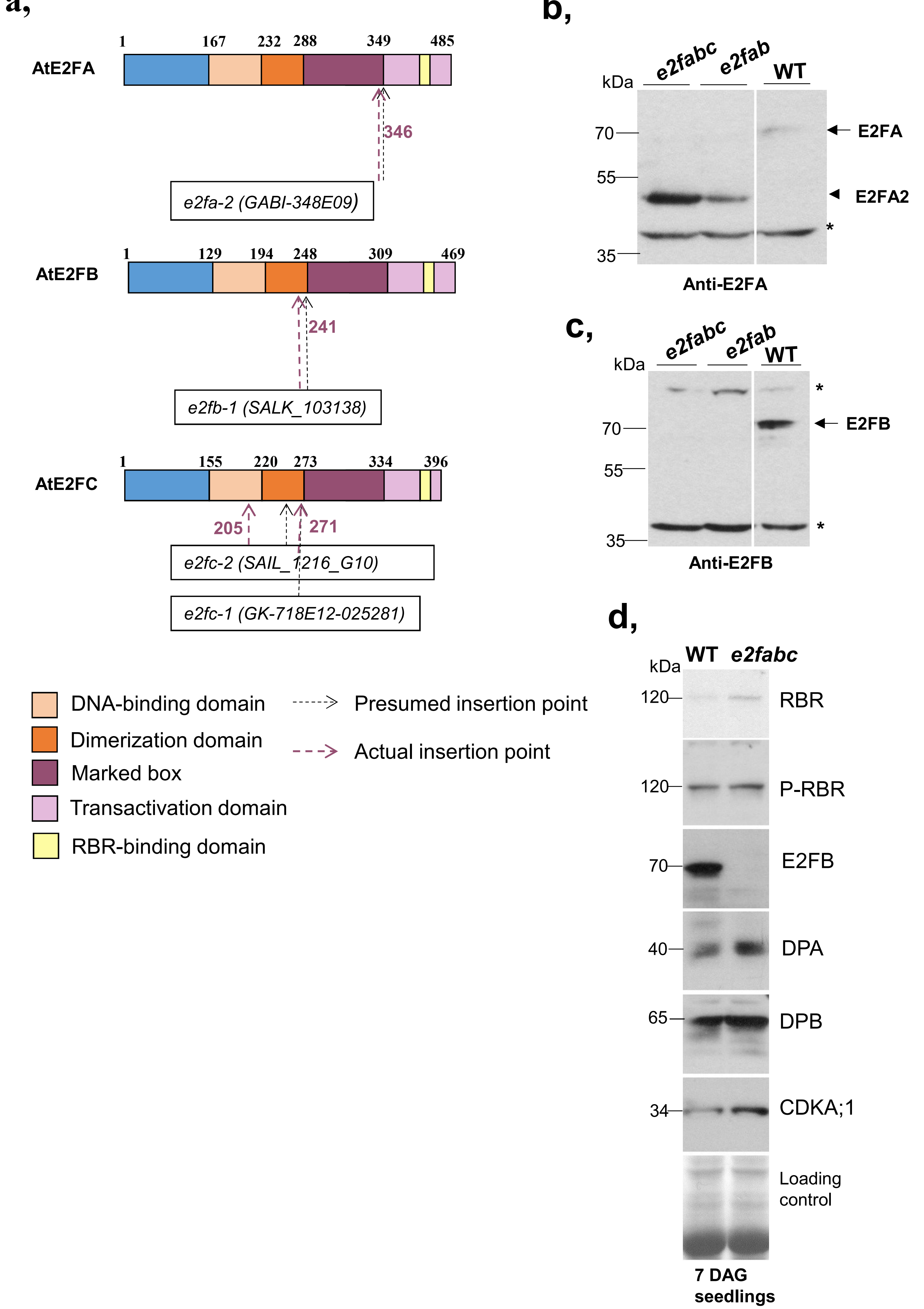

Supplementary Fig. 1 Characterization of E2F mutant lines used in this work.

a Position of the T-DNA insertions in mutant lines used in this study. In each mutant the exact position of the insertion was verified by sequencing the flanking regions and the presumed and the actual insertion points are shown by black and purple arrows, respectively. For all genes, the regions encoding each protein domain are highlighted with different colours.

b, c Accumulation of E2FB is undetectable in *e2fab*, and *e2fabc* mutants using a C-terminal specific anti-E2FB antibody in western blot whereas the N-terminal specific anti-E2FA antibody recognized a truncated version of the E2FA protein in the *e2f* mutants (marked as E2FA2 – Leviczky et al., 2019). Arrow shows the full length E2FA and E2FB proteins, while arrowhead marks the truncated E2FA (E2FA2) and the star indicates an aspecific band recognized by the anti-E2FB antibody.

d The accumulation level of RBR, phosphorylated RBR (P-RBR^{911Ser}), CDKA;1, DPA and DPB proteins was comparable in the *e2fabc* with the WT or even slightly enhanced (description of these antibodies are found in the Methods section). Seedlings at 7DAG were used in Western blot assay. Molecular weight markers are indicated at the left side.

Supplementary Fig. 2

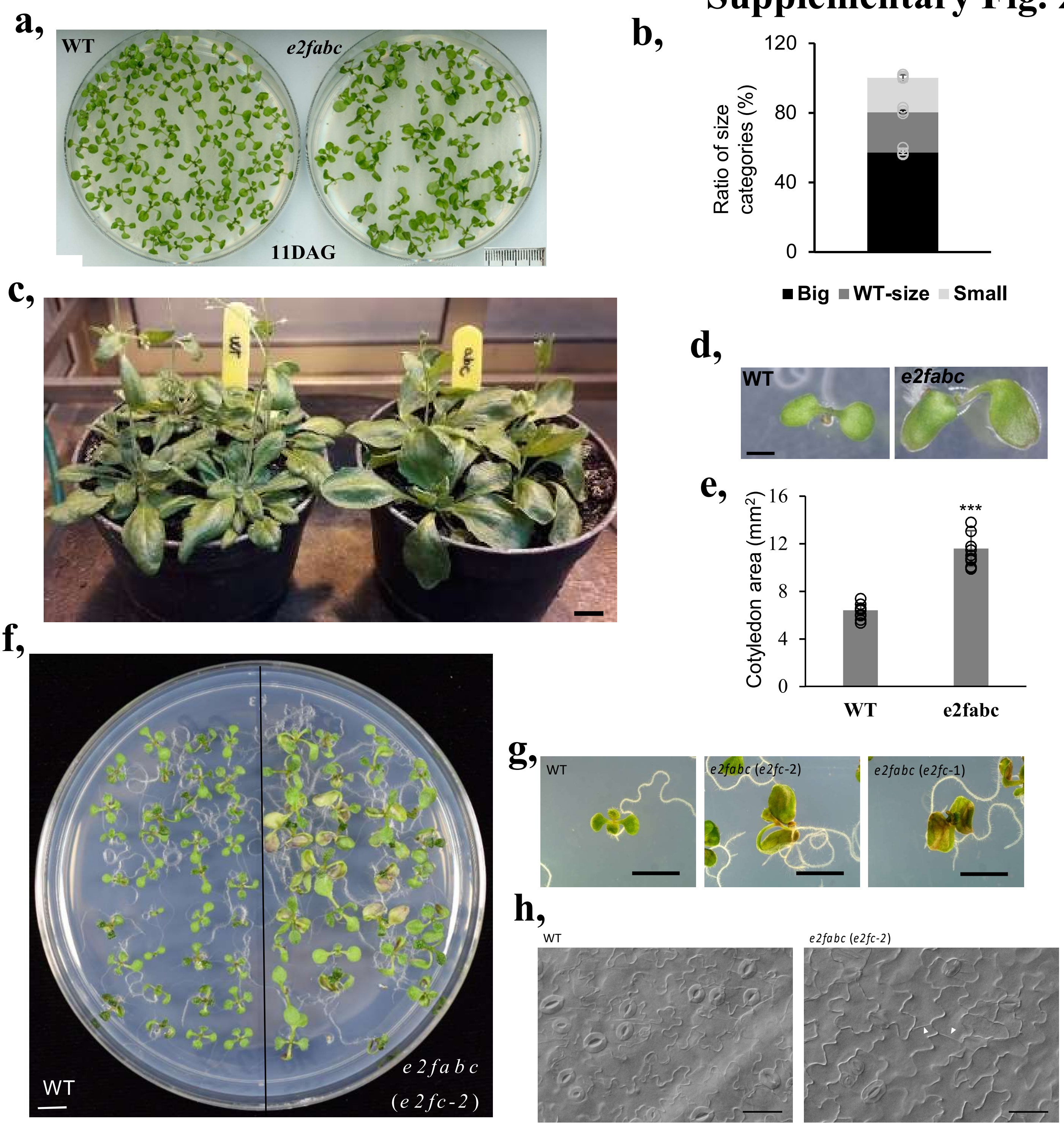

Supplementary Fig. 2 ***E2fabc*** **mutants are enlarged compared to the WT.**

a Representative phenotypes of plantlets at 11 DAG grown on agar medium. **b** Distribution of *e2fabc* plantlets between big, WT-size and small categories determined at 11 DAG. **c** Picture of WT and *e2fabc* plants with fully expanded rosettes grown for 23 days on soil (Bar: 1 cm). **d, e** Cotyledons of *e2fabc* mutants are enlarged. **d** Pictures of plantlets at 7 DAG (Bar: 4 mm), (**e**) Graph showing cotyledon area in plantlets at 7 DAG. Data are average +/- standard deviation (n=3 biological replicates, N=10 samples in each). ***$P \leq 0.001$, denotes statistically relevant differences (two-tailed, paired *t*-test between the WT and the mutant). **f-h** Young seedlings of *e2fabc-2* triple mutant are enlarged. **f** Pictures of plantlets at 9 DAG (Bar: 0,5cm), (**g**) Higher magnification of seedlings at 9 DAG, scale bar = 5mm (**h**) DIC image of differentiated pavement cells in the leaf epidermis of the triple *e2fabc-2* line show newly synthesised cell walls indicating cell proliferation. Scale bars = 20 µm.

Supplementary Fig. 3

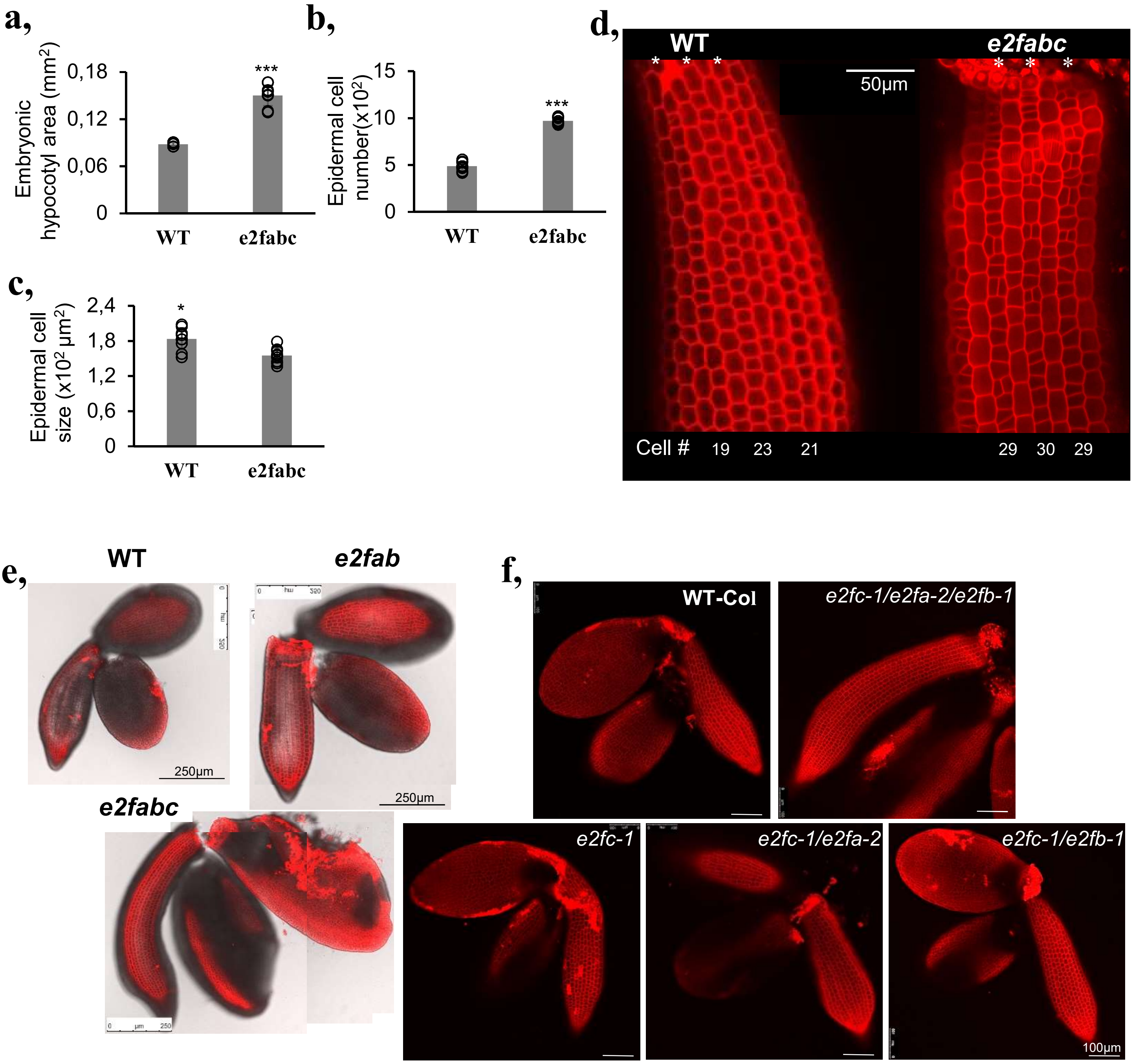

Supplementary Fig. 3 Excessive proliferation is only apparent in the triple *e2fabc* mutant embryos. a-c Hypocotyls of *e2fabc* embryos are enlarged and consist of more numerous and smaller cells compared to the WT. **a** Embryonic hypocotyl area, (**b**) Epidermal cell size, (**c**) Epidermal cell number. **d-f** Representative confocal microscopy image showing the hypocotyl epidermis and dissected embryos after propidium iodide (PI) staining. **d** Comparison of hypocotyl epidermis of *e2fabc* and the WT control. Asterisks show longitudinal cell files where extra cell divisions were observed in the *e2fabc* mutant but not in the WT. **e** The e*2fabc* triple embryo is even larger than the enlarged *e2fab* double mutant embryo (Bar: 250 µm). **f** Neither *e2fc-1* nor double *e2fac/e2fbc* mutant embryos did show excessive proliferations in their hypocotyl epidermis, and they are more similar in size with WT control embryo (Bar: 100µm). For all graphs, data are average +/- standard deviation, (n=3 biological repeats, N=10 samples in each), **P*≤0.05, ***P*≤0.01 denotes statistically relevant differences (two-tailed, paired *t*-test between the WT and the mutant).

Supplementary Fig. 4

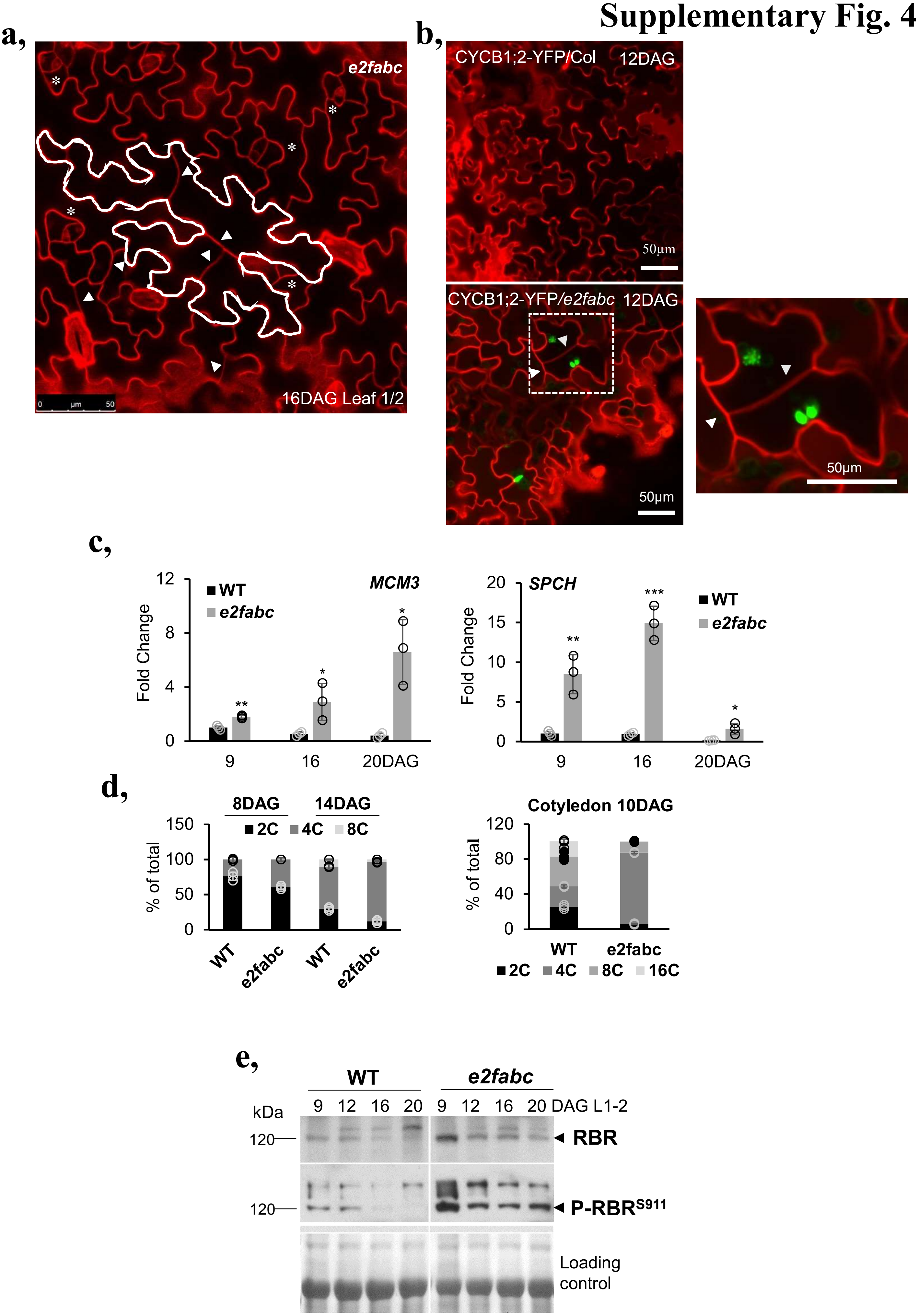

Supplementary Fig. 4 Excessive cell proliferation is enhanced in *e2fabc* mutants.

a The *e2fabc* mutant leaves display extra cell division events in puzzle-shaped differentiated epidermal cells. An example of a large dividing puzzle-formed pavement cell is outlined in white. Arrowheads show straight cell lines indicative for multiple extra cell divisions. Asterisks mark dividing small meristemoid-like cells. Picture of leaf epidermis was taken at 16DAG. Bar: 50 µm.

b The CYCB1;2-YFP^{NLS} signal was not detectable in the epidermal pavement cells of the WT-Col background but in the leaf of *e2fabc* mutant at 12DAG. Elongated epidermal pavement cells with a straight cell wall inside is marked by a white box containing YFP signal in their nuclei indicating ongoing mitosis and it was zoomed on the right side. Arrowheads indicate newly formed straight cell walls. Bar: 50 µm.

c The cell cycle gene *MCM3* and the stomata development gene *SPCH* are overexpressed in the first leaf pairs of *e2fabc* mutants compared to the WT at 9, 16, 20 DAG. Expression of the two genes was quantified by qRT-PCR, and values represent fold change levels normalised to the relevant transcript levels of the WT at 9DAG, which was set arbitrarily at 1. n=3 biological repeats. Error bars indicate the +/- SD. *$P \leq 0.05$, **$P \leq 0.01$, ***$P \leq 0.001$; indicate statistical significance two-tailed, paired *t*-test between the wild type and the e2fabc at a given time point. Abbreviations and primer sequences are listed in Supplementary Table 1.

d Percentage of DNA ploidy levels were determined by flow cytometry in first leaf and cotyledon samples at the indicated time points (DAG).

e In immunoblot assay, phosphorylation on the conserved Ser^{911} site in the RBR protein (P-RBR) remained at high level in the triple *e2fabc* mutant in comparison to the WT during leaf development at 9, 12, 16, 20 DAG. Arrowheads indicate the postions of RBR and P-RBR proteins. Molecular weight size is indicated on the left.

Supplementary Fig. 5

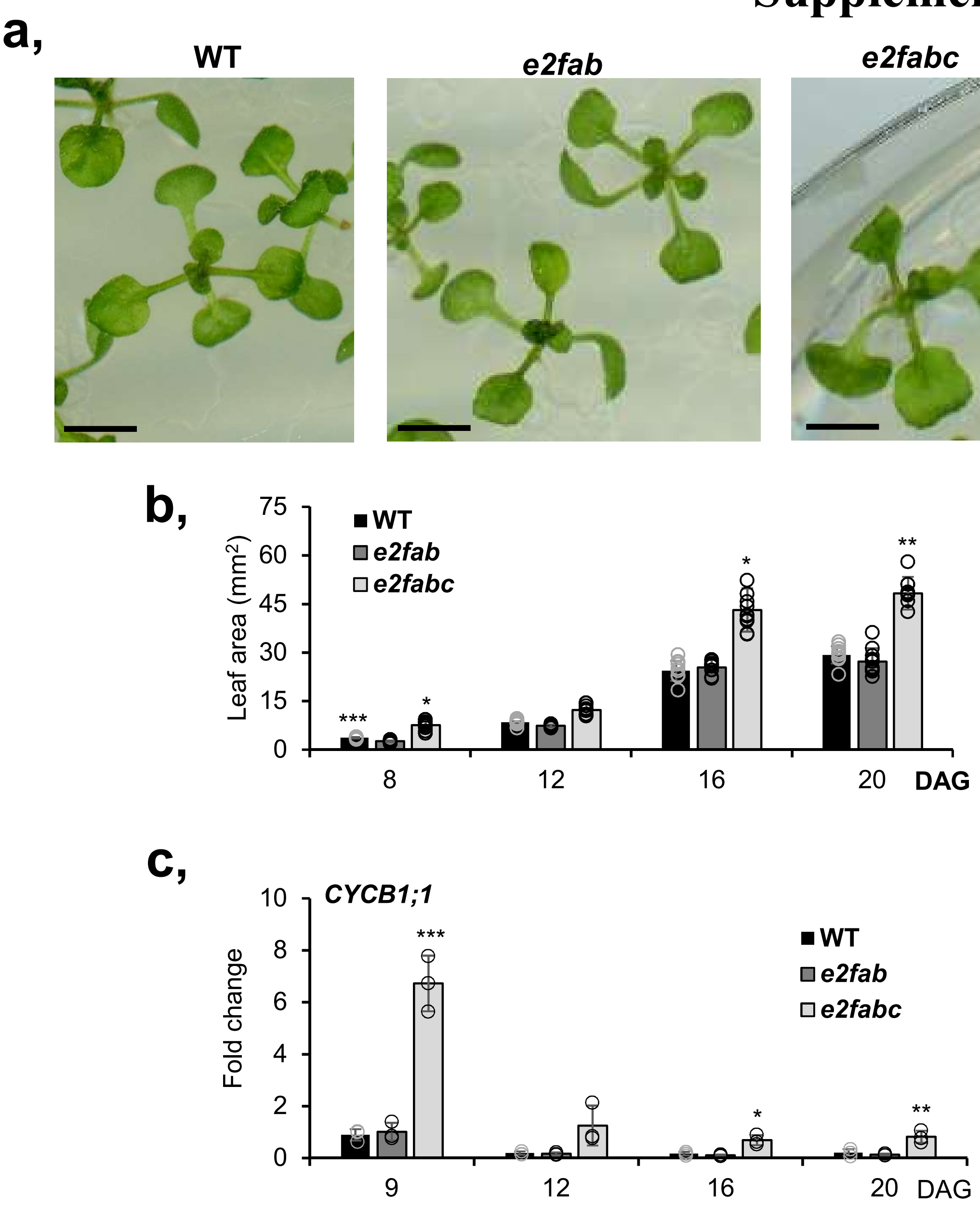

Supplementary Fig. 5 Leaf size and expression of the mitotic *CYCB1;1* was not affected in the double *e2fab* mutant but in the triple *e2fabc*.

a Representative pictures of seedlings of WT, *e2fab* double and *e2fabc* triple mutant lines were taken at 13DAG. Bar: 3mm.

b Quantification of leaf area (first leaf pair) during leaf development of WT, *e2fab* and *e2fabc* lines at 8,12,16 and 20 DAG. Data are average +/- standard deviation n=3 biological replicates, N=10 samples in each. *P<0,05; ***P*< 0.01; *** *P*< 0,001 (two-tailed, paired t-test between the WT and the two mutants at a given time point).

c The mitotic *CYCB1;1* shows significantly elevated expression in *e2fabc* mutant compared to the wild-type and to the *e2fab* double mutant during leaf development at 9, 12, 16 and 20 DAG. Expression was monitored by qRT-PCR. Values represent fold changes relative to average expression levels of two reference genes (*UBC18* and *ACTIN*) and normalised to the value of the relevant transcript of the wild type at 9DAG, which was set arbitrarily at 1. Data are means +/- sd., n=3 biological repeats. ***P*< 0.01;, *** *P*< 0,001 (two-tailed, paired t-test between the WT and the two mutants at a given time point). Abbreviations and primer sequences are listed in Supplementary Table 1.

Supplementary Fig. 6

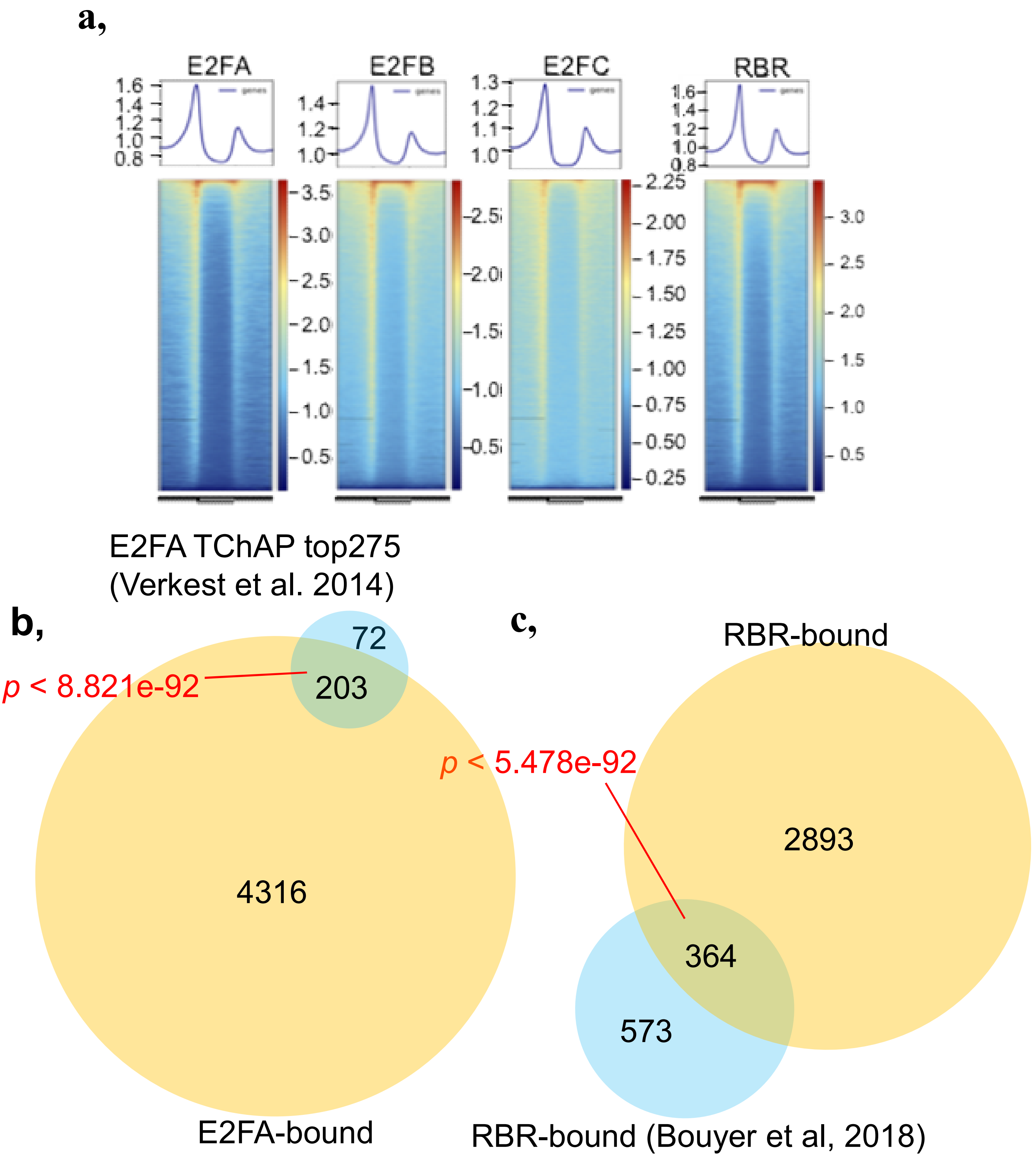

Supplementary Fig. 6 Quality control of the ChIP-seq analysis

a Metaplot and heatmap showing the binding profiles of E2FA, E2FB, E2FC and RBR. Plots are centred on target genes.

b comparison of E2FA targets identified in this work, and the top 275 genes defined in previous TChAP experiments (Verkest et al., 2014).

c comparison of RBR targets identified in this work, and those targets reported by (Bouyer, Heese et al., 2018). For both comparisons, overlaps obtained are significantly greater than what would be expected by chance, *P*-values are indicated in red (Fisher exact test).

Supplementary Fig. 7

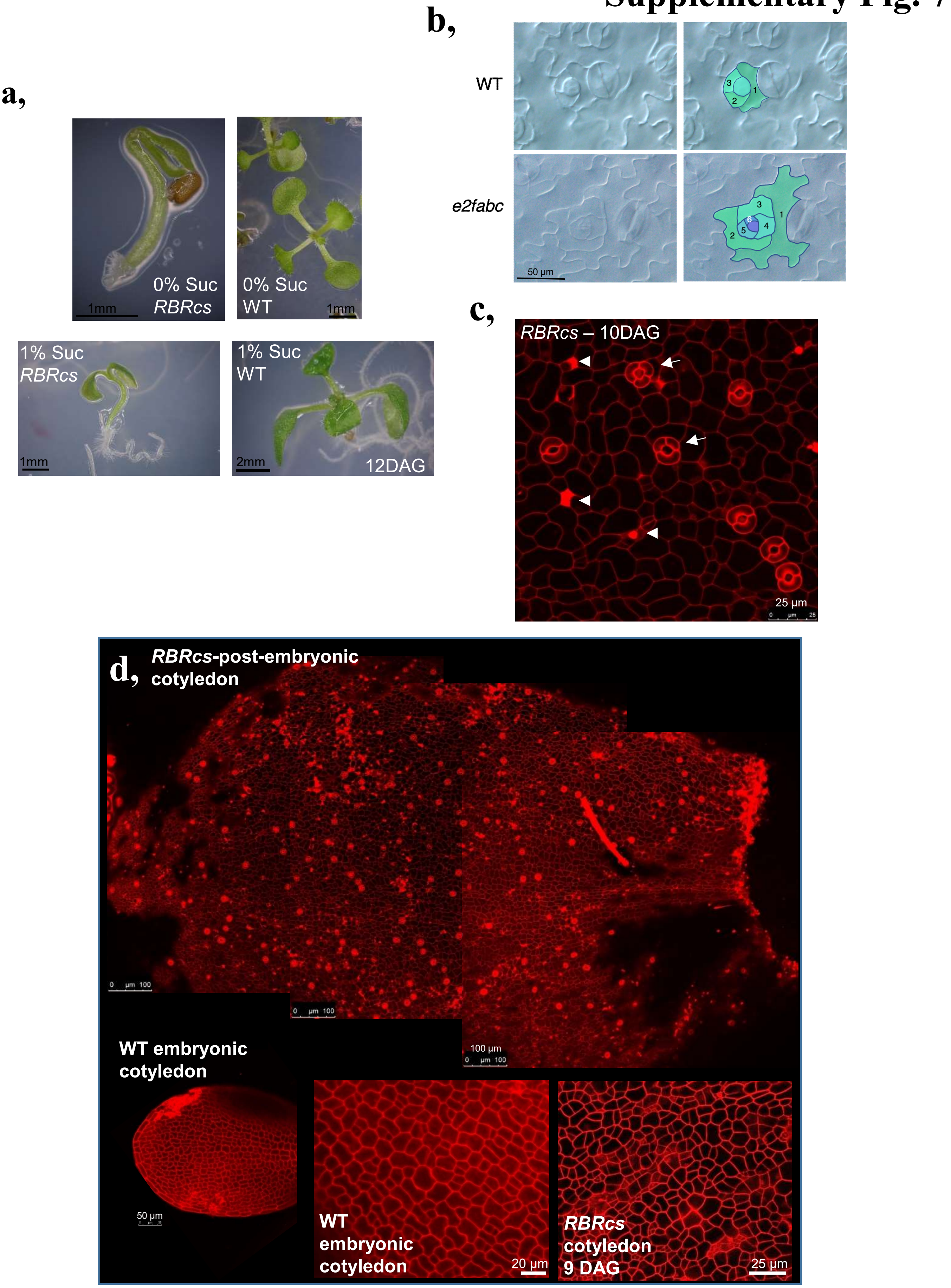

Supplementary Fig. 7 Comparison of cellular phenotypes observed in the epidermis of *e2fabc* and *RBRcs* lines.

a All mutant seedlings with reduced RBR levels had identical phenotypic abnormalities as reported earlier (Gutzat et al., 2011). On sucrose free medium the root of the *RBRcs* seedlings did not grow, and cotyledons were closed (12DAG), while in the presence of sucrose roots grow and cotyledons were open but they never produced expanding first leaf pairs, and their growth was strongly arrested in comparison to the WT at the same age. Scale bars are indicated.

b DIC image of a stomata meristemoid (top) and cartoon highlighting reiterated cell division events in the *e2fabc* leaf in comparison to the WT. Bar: 50 µm.

c-d PI stained cotyledons of *RBRcs* seedlings (at 9 DAG) and WT embryos. The obtained images are highly similar, showing that epidermal cells of *RBRcs* remain embryonic-like. Arrows show stomata consisting of three cells, while arrowheads indicate dead cells in d.

Supplementary Fig. 8

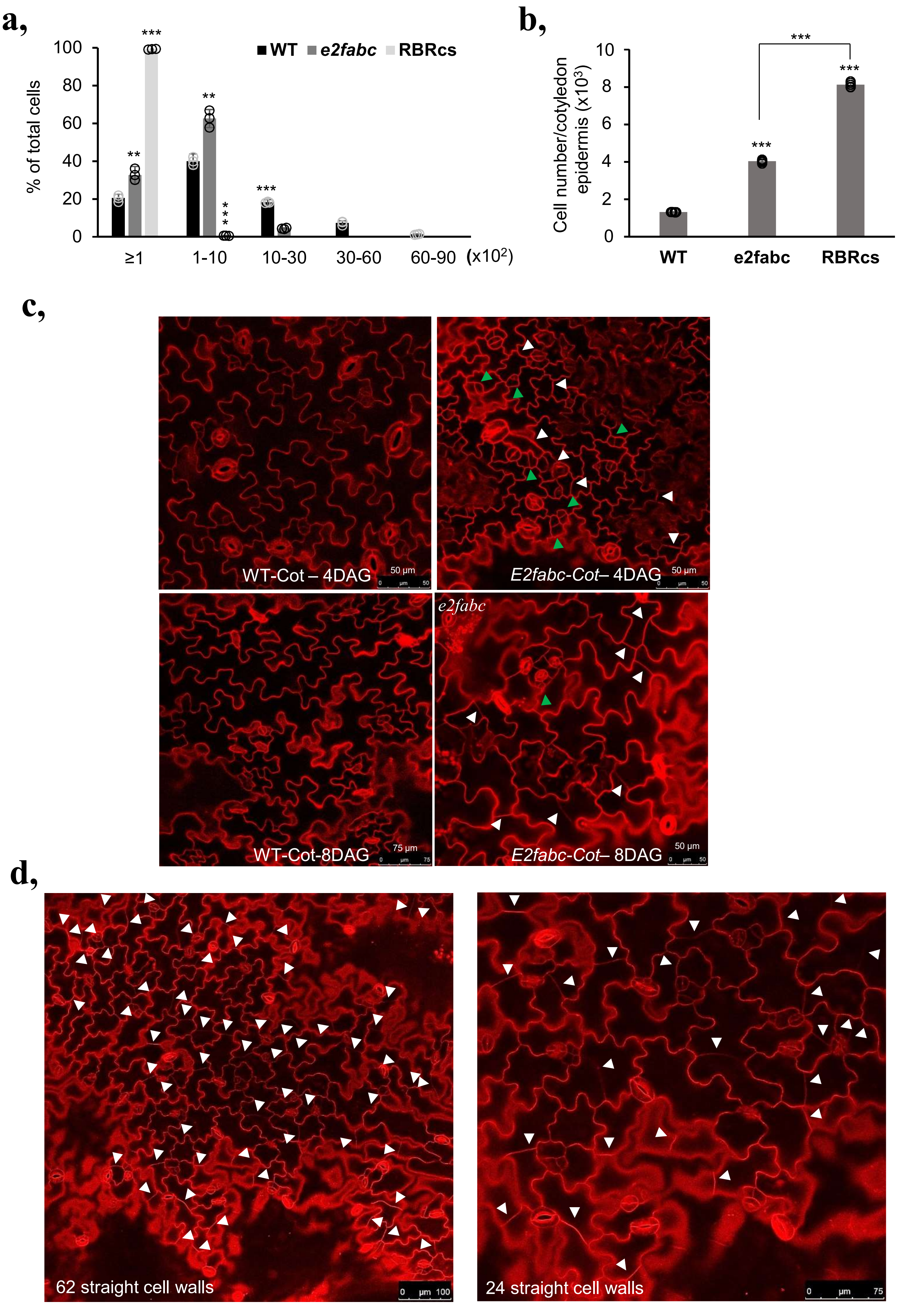

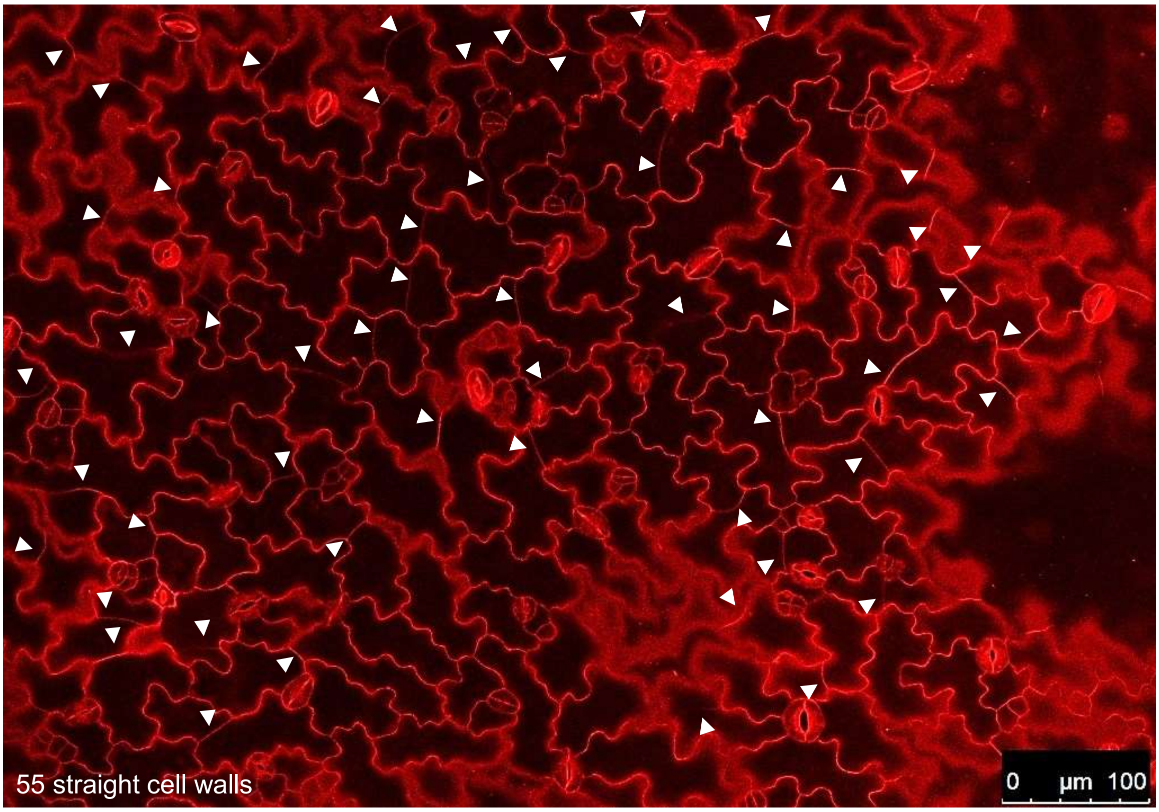

Supplementary Fig. 8 Excessive cell proliferation in *e2fabc* mutants and *RBRcs* lines

a Cell size distribution in the cotyledon epidermis of *e2fabc* and *RBRcs* lines at 4DAG is shifted towards the smaller cell sizes. n=3 biological repeats, N=10 samples in each. ≥400 cells were measured using ImageJ. ***P*≤0.01, ****P*≤0.001, significant difference in the transgenic lines compared to the WT using Student t-test.

b Cell number is increased in the cotyledon epidermis of *RBRcs* lines and to a lesser extent in *e2fabc* lines. ****P*≤0.001; indicate statistical significance (two-tailed, paired *t*-test between the WT and the mutants, and between the two mutants).

c Representative images of propidium iodide (PI) stained cotyledon epidermis of WT and *e2fabc* mutants at 4 and 8DAG as indicated. Green arrowheads point at dividing small meristemoid-like cells, and white arrowheads show at extra cell division events occurring in differentiated cells. Scale bar: 50 or 75 μm as indicated.

d Elongated pavement cells keep dividing in the *e2fabc* cotyledon epidermis at 6DAG judged by the frequent appearence of straight cell walls (indicated by arrowheads in the confocal microscopic images). Their exact numbers were also presented. Scale bars are indicated.

Supplementary Fig. 9

a,

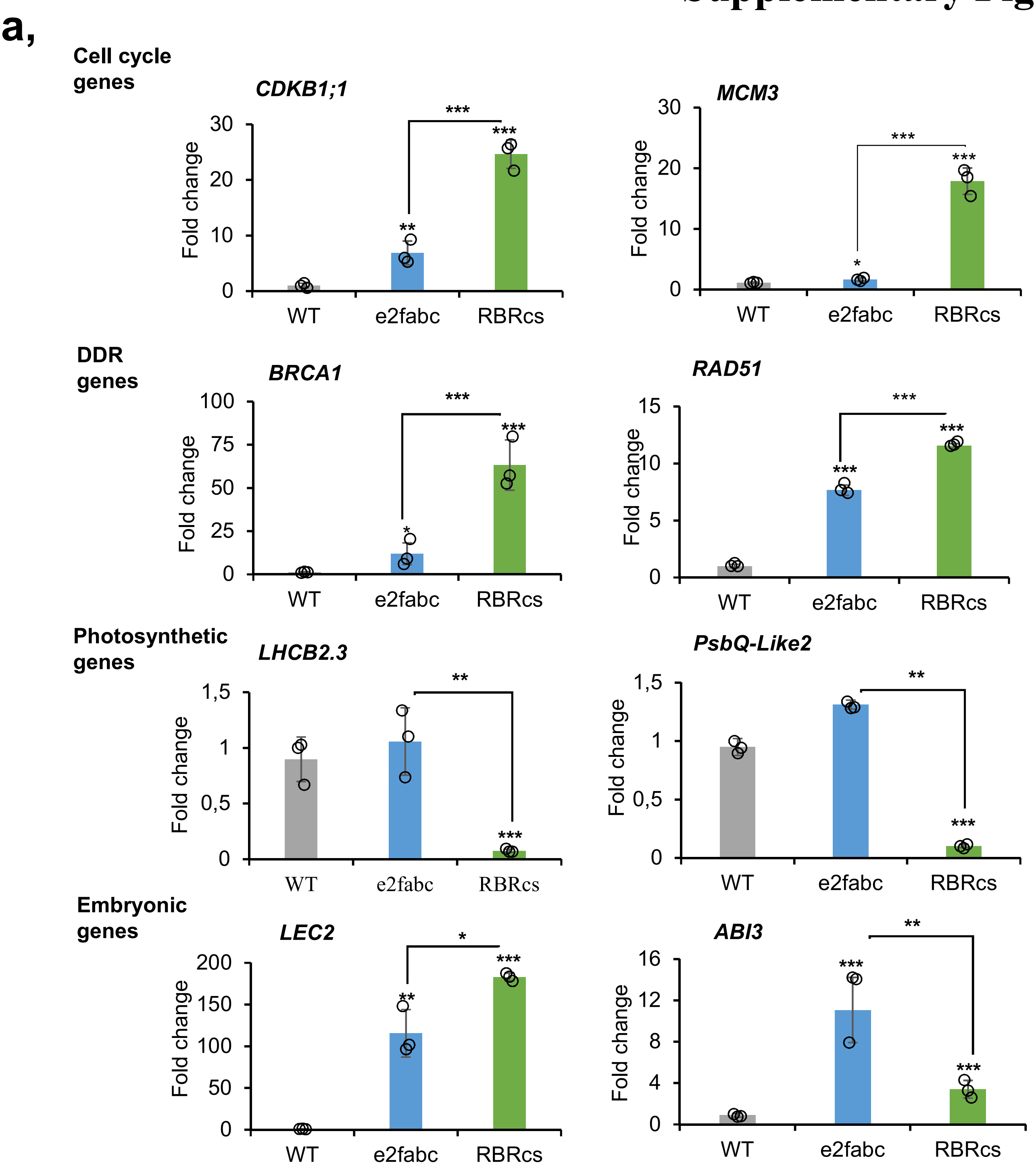

b,

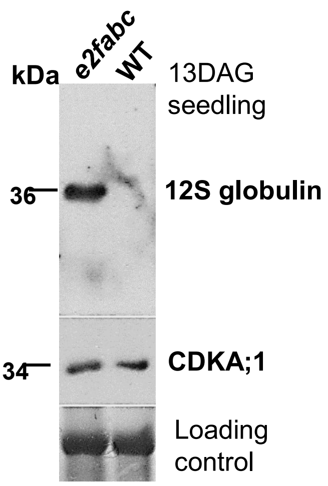

Supplementary Fig. 9 RT-qPCR quantification of marker genes in *e2fabc* and *RBRcs* lines

a Cell cycle and DNA repair genes are highly induced in *RBRcs* line, and to a lesser extent in *e2fabc* mutant. By contrast, differentiation-related genes are specifically repressed in *RBRcs*, but not in *e2fabc* mutants. Values represent fold changes relative to average expression levels of two reference genes (*UBC18* and *ACTIN*) and normalised to the relevant transcript levels in the WT, which was set arbitrarily at 1. n=3 biological repeats. Error bars indicate the SD. *$P<0.05$, **$P\leq 0.01$, ***$P\leq 0.001$ indicates statistical significance determined using two-tailed, paired *t*-test between the WT and the mutants and between the mutants. Abbreviations and primer sequences are listed in Supplementary Table 1.

b Seed storage 12S globulin accumulated in the *e2fabc* mutant seedlings 13 DAG but not in the WT at the same age. CDKA;1 proteins were at comparable levels in both of these lines. Ponceau-S stained proteins on the same membrane were used as loading control.

Supplementary Fig. 10

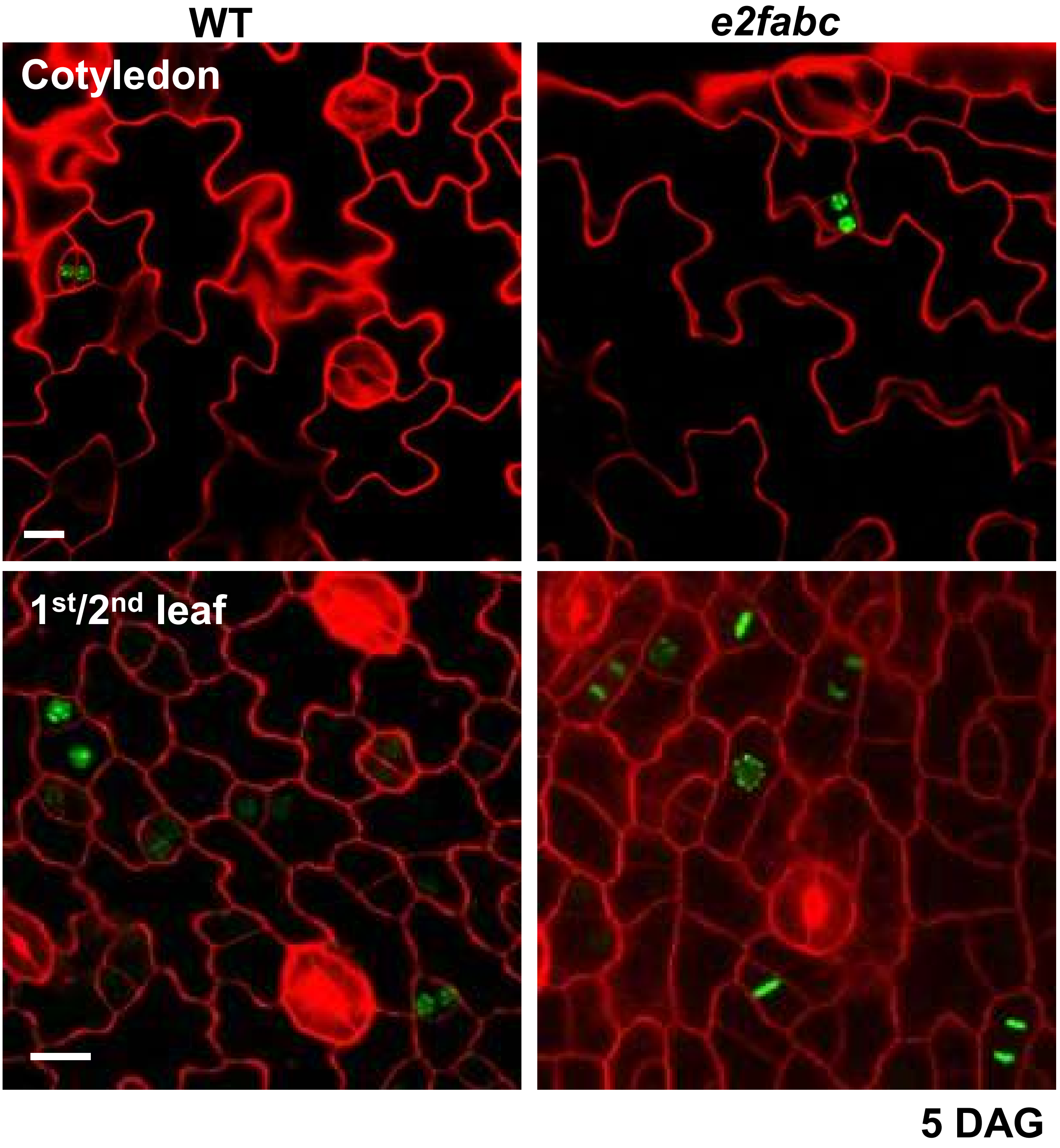

Supplementary Fig. 10 Expression of the cell division marker CYCB1;2 is enhanced in *e2fabc* mutants.

Cotyledons and first leaves of WT and *e2fabc* mutants expressing a YFP-tagged version of CYCB1;2 were stained with propidium iodide (PI). Expression of the cell division marker was low in cotyledons of both genotypes, but markedly increased in the first leaves of *e2fabc* mutants compared to the WT. This CYCB1;2-YFP protein accumulated throughout mitosis including cycling cells in anaphase and telophase. Scale bars = 10 µm.

Supplementary Fig. 11

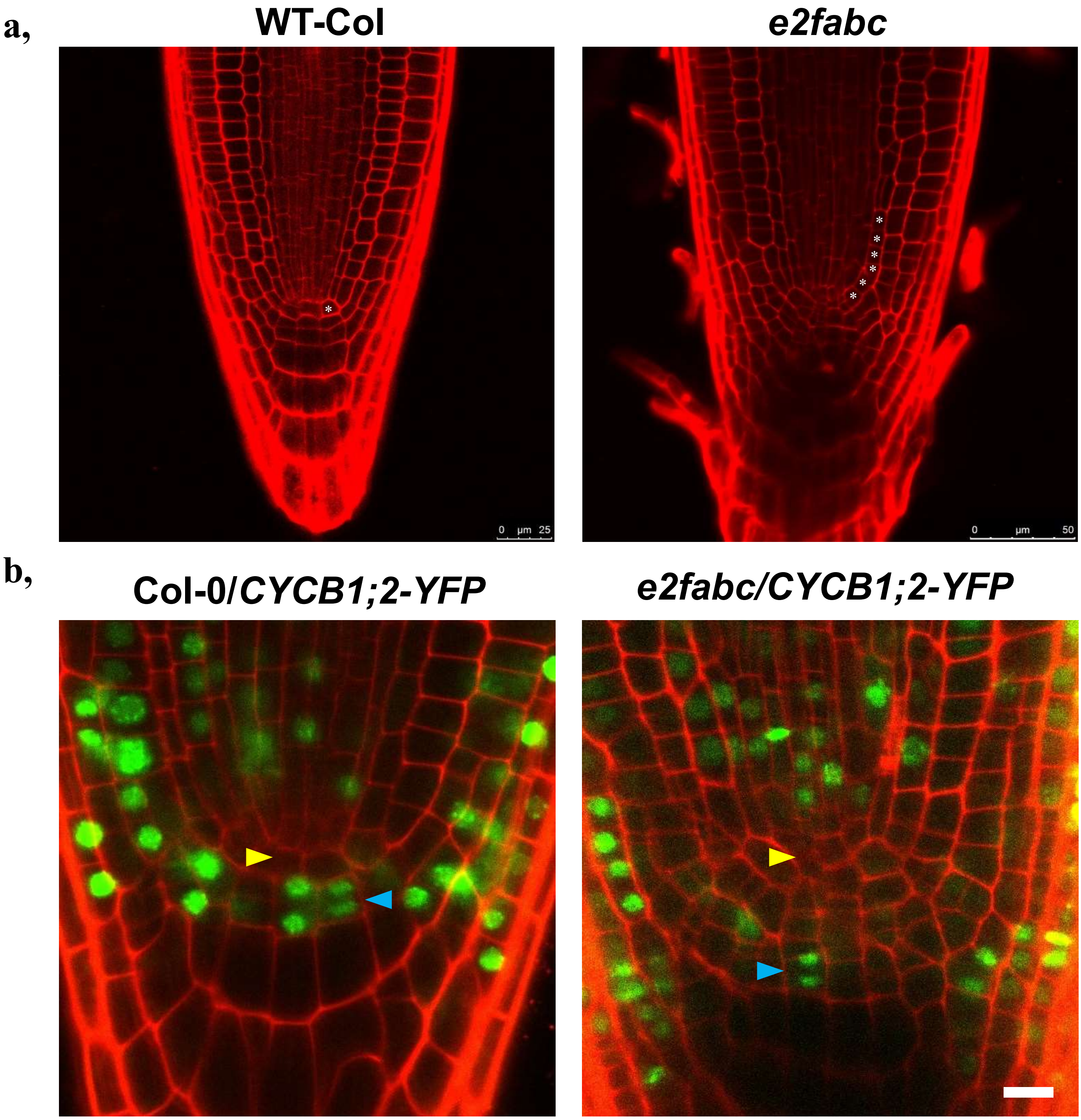

Supplementary Fig. 11 Enhanced cell proliferation of stem cells in *e2fabc* root meristems

a Confocal microscopy images showing that *e2fabc* mutants display an increased number of cortex/endodermis initials (marked inside by asterisks) compared to the WT. Scale bar is indicated.

b expression of the CYCB1;2-YFP marker in the root meristem of WT and *e2fabc* mutants. Blue arrowheads indicate cell division events in the columella initials. One such event is observed in supernumerary columella initials of the *e2fabc* mutant. Scale bar: 10 µm.

Supplementary Fig. 12

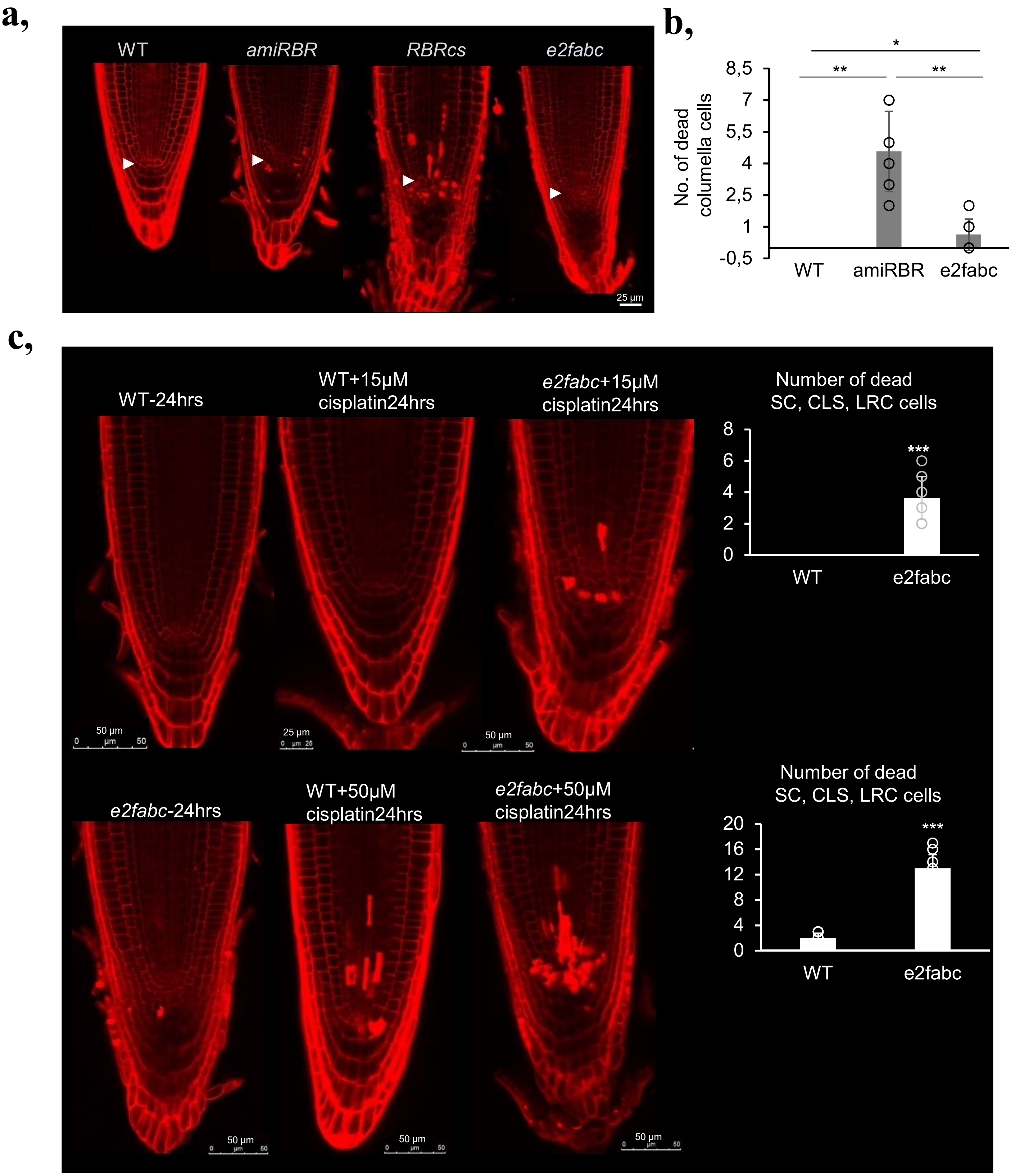

Supplementary Fig. 12 Loss of quiescence in RBR loss of function and *e2fabc* lines affects meristem maintenance

a Spontaneous cell death is observed in meristems of RBR loss of function lines but not in *e2fabc* mutants in control conditions. Root tips of seedlings at 4 DAG were stained with propidium iodide (PI) and imaged under a confocal microscope. Arrowhead marks the position of QC cells.

b Quantification of cell death in the columella of *amiRBR* and *e2fabc* mutants. Data are average +/- SD, n=3 biological replicates, N=8 in each. *$P \leq 0.05$, **$P \leq 0.01$; indicate statistical significance using Student's *t*-test comparing *amiRBR* and *e2fabc* to WT and *amiRBR* to the *e2fabc*.

c *e2fabc* mutants are hypersensitive to cisplatin. Plantlets of WT and *e2fabc* mutants at 5 DAG were treated with the indicated dose of cisplatin for 24h, and stained with PI prior to confocal imaging. Cell death was more strongly induced by both doses of cisplatin in the *e2fabc* mutant than in the WT. Data are average +/- SD, n=3 biological replicates, N=10 in each. ***$P \leq 0.001$ indicate statistical significance using Student's *t*-test comparing *e2fabc* to WT.

Supplementary Fig. 13

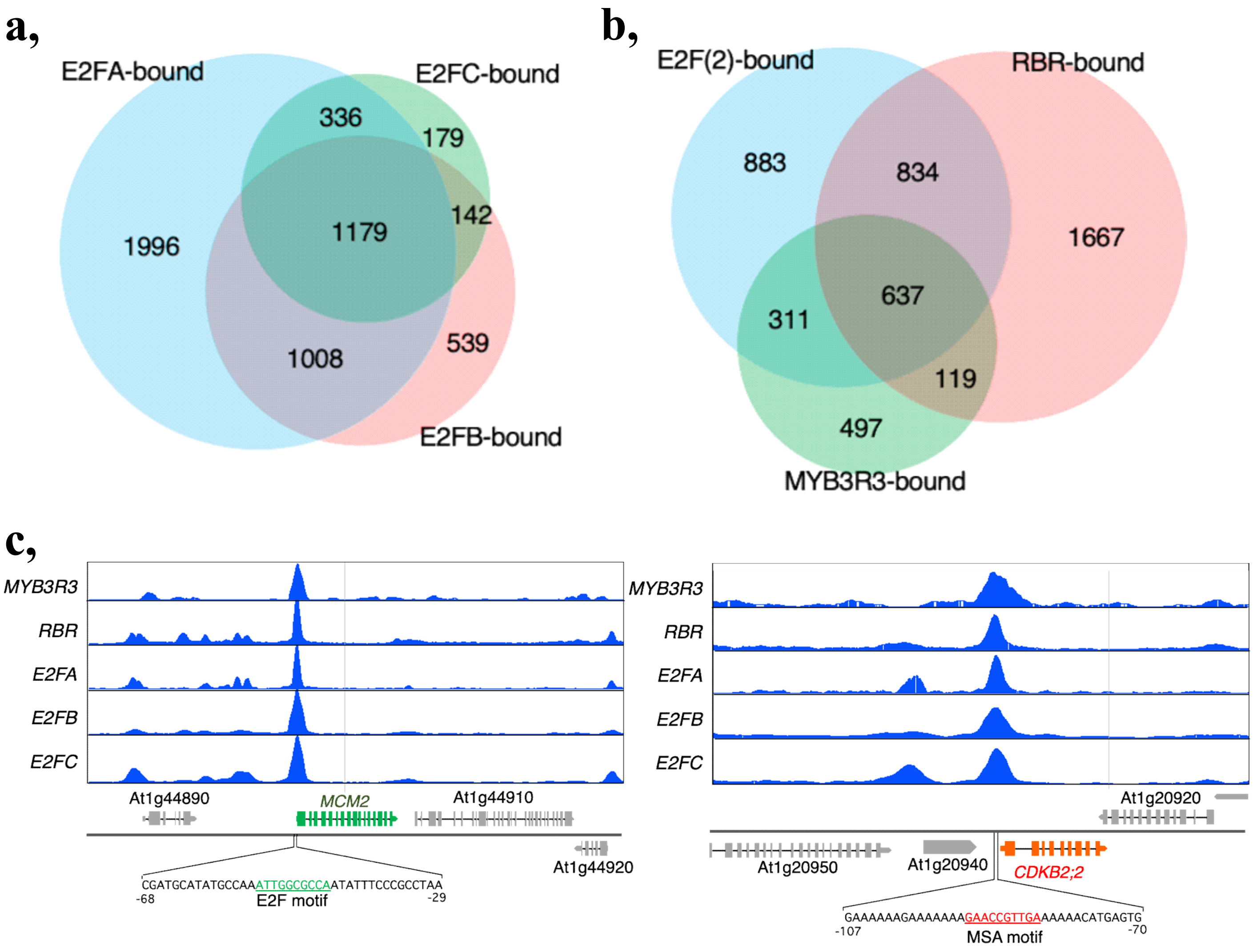

Supplementary Fig. 13 Overlap between E2F and MYB3R targets

a Venn diagram showing the overlap between the best targets for each E2F (FC>3 *P*<0.01).

b Venn diagram showing the overlap between genes bound by at least two E2F factors E2F(2) and the best RBR and MYB3R3 targets (FC>3 *P*<0.01).

c Screenshots illustrating that E2FA,B,C, RBR and MYB3R3 are found at the same position on their common targets. Here we chose *MCM2*, on which all factors are found in the region harbouring the canonical E2F binding site, and *CDKB1;2*, on which all 4 factors are found in the region harbouring the canonical MSA binding site.

GI	NAME	FWD	REV	PROD. SIZE
AT1G80080	TMM	GGACGTGAAGCATCTAAGCGA	TCAGCTTTCTCCTCATCCTCC	101
AT2G37560	ORC2	ACCATGTCAACGCTCCATTA	TCGACATTGTATGGTGCAAA	100
AT1G28300	LEC2	GCAAGAATCTCTACTTCGCC	CTTCCTCTTCGTCTCTTGGT	129
AT5G11510	MYB3R4	AATCGCTTGAGAAAGTAGACC	AGTAGACAGGACTGGCTTACC G	140
AT3G09370	MYB3R3	AGTATCACCTACTCATAGGTAC	AGCTCTTGCCTTTAAACGTGTC	125
AT3G01440	PQL2/PnsL3	ACAAGAACAGAGGCTGACACC	CGGTTTTACGACTCTCGGGT	122
AT3G27690	LHCB	CAAGTCTACTCCTCAGAGCA	CGTAGTCTCCAGGGTATTCTC	110
AT4G21070	BRCA1	TCATGGGAGATTTCGAGCTT	ATTTAGCCAAGGCTTCAGCA	195
AT5G20850	RAD51 var 88 &92	TCCCTGTCGAACAGCTTCAG	CCTGTCTCTGAGCATGGAGC	225
AT3G54180	CDKB1;1	TCTGTTGGTTGTATCTTTGCTGA	CATTGCTGCTCAGTTGGTGT	119
AT3G24650	ABI3	GGCAGGGATGGAAACCAGAAA AGA	GGCAAAACGATCCTTCCGAGG TTA	94
AT5G53210	SPCH	GCTGCTCTTGAAGATTTGGCT	CACTCAATTCCAATCTTGATGG TG	101
AT1G08560	KNOLLE	GCTCGGATCGAACAGTACCA	CGCCTTCCTCAAACCAGACA	111
AT5G46280	MCM3	TGGGCAGCACATGAGGAC	CACTTTGTTATCATCTTGCAGT TT	148
AT5G42990	UBC18	ACAGCAATGGACATATTTGTTTA GA	TGATGCAGACTGAACTCACTG TC	78
AT3G18780	ACTIN	GACCTTTAACTCTCCCGCTATG	CAGAATCCAGCACAATACCG	92
AT4G37490	CYCB1;1	AGCGAAAGGAAGAAACCGTCA	ATCCTCCACAAGAAGCGTGG	139

Supplementary Table 1 List of primers and their sequences used for qRT-PCR analysis.

In the first row from left to right, gene identification (GI), gene's name, forward (FWD) and reverse (REV) sequences, and the product size of the PCRs in base pair (PROD. SIZE).

Supplementary Fig. 14

Fig. 4a

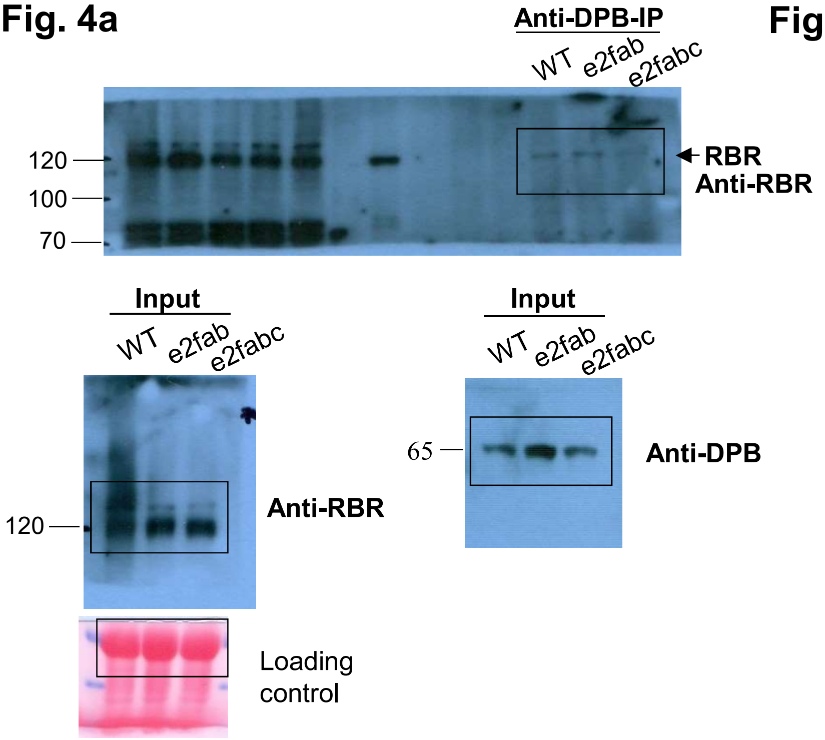

Fig. 4b

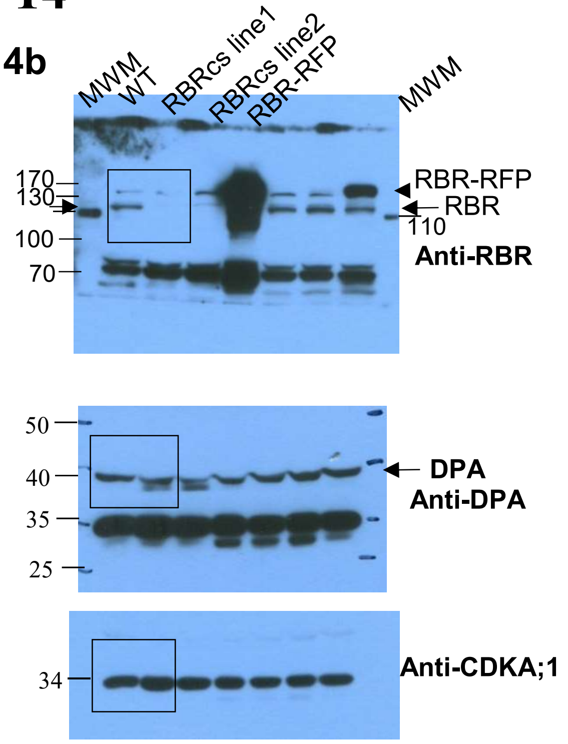

a,

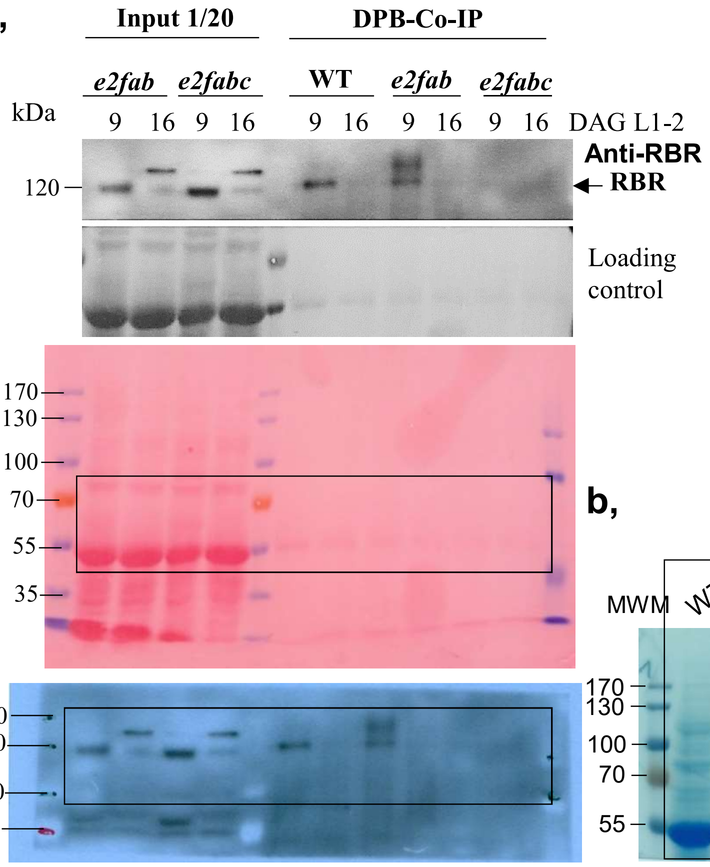

b,

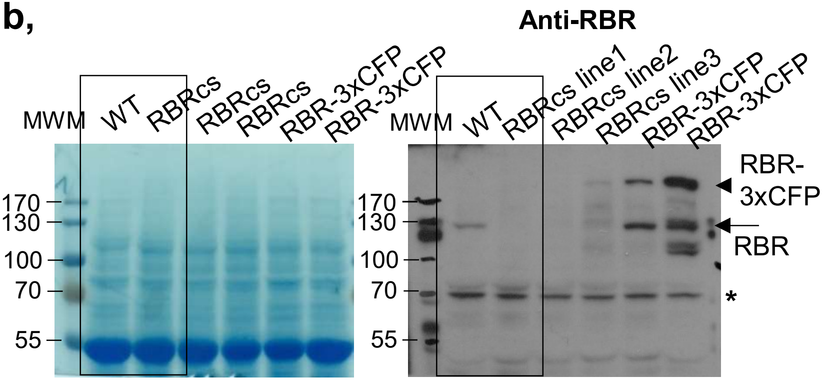

Supplementary Figure for Fig.4a and b. ***a*** DPB could form complex with RBR at comparable level in the WT and in the *e2fab* double mutant leaf during its development while hardly any RBR was precipitated by the DPB antibody from the triple *e2fabc* mutant leaf. 1/20 were loaded to the input samples. Arrow indicates the position of RBR protein. ***b*** RBR protein was not detectable in 7DAG old seedlings of the *RBRcs* mutant lines. Arrow shows RBR. RBR antibody recognized a protein at 70kDa presumably a degradation product of RBR (marked by an asterisk). Molecular weight markers are indicated on the left side. Ponceau-S (pink) and Coomassie (blue) stained membranes are presented (in *a* and *b*, respectively).

Supplementary Fig. 1b

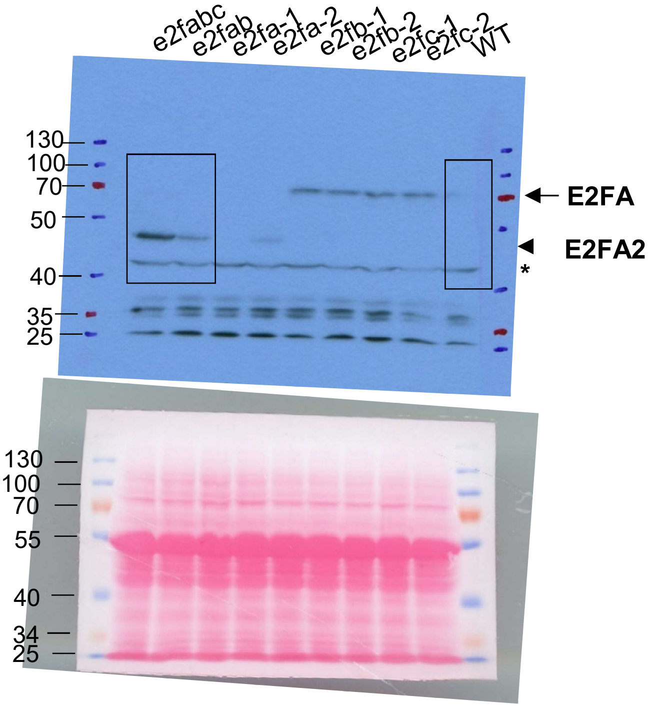

Anti-E2FA

Supplementary Fig. 1c

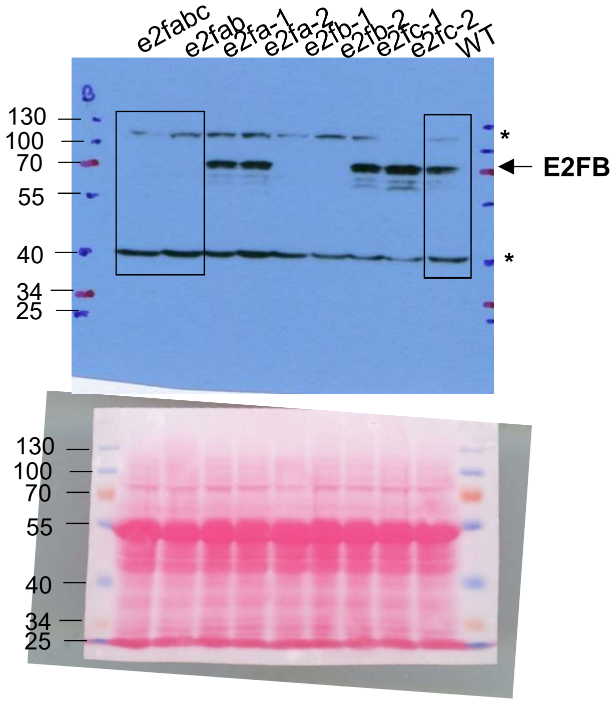

Anti-E2FB

Supplementary Fig. 1d

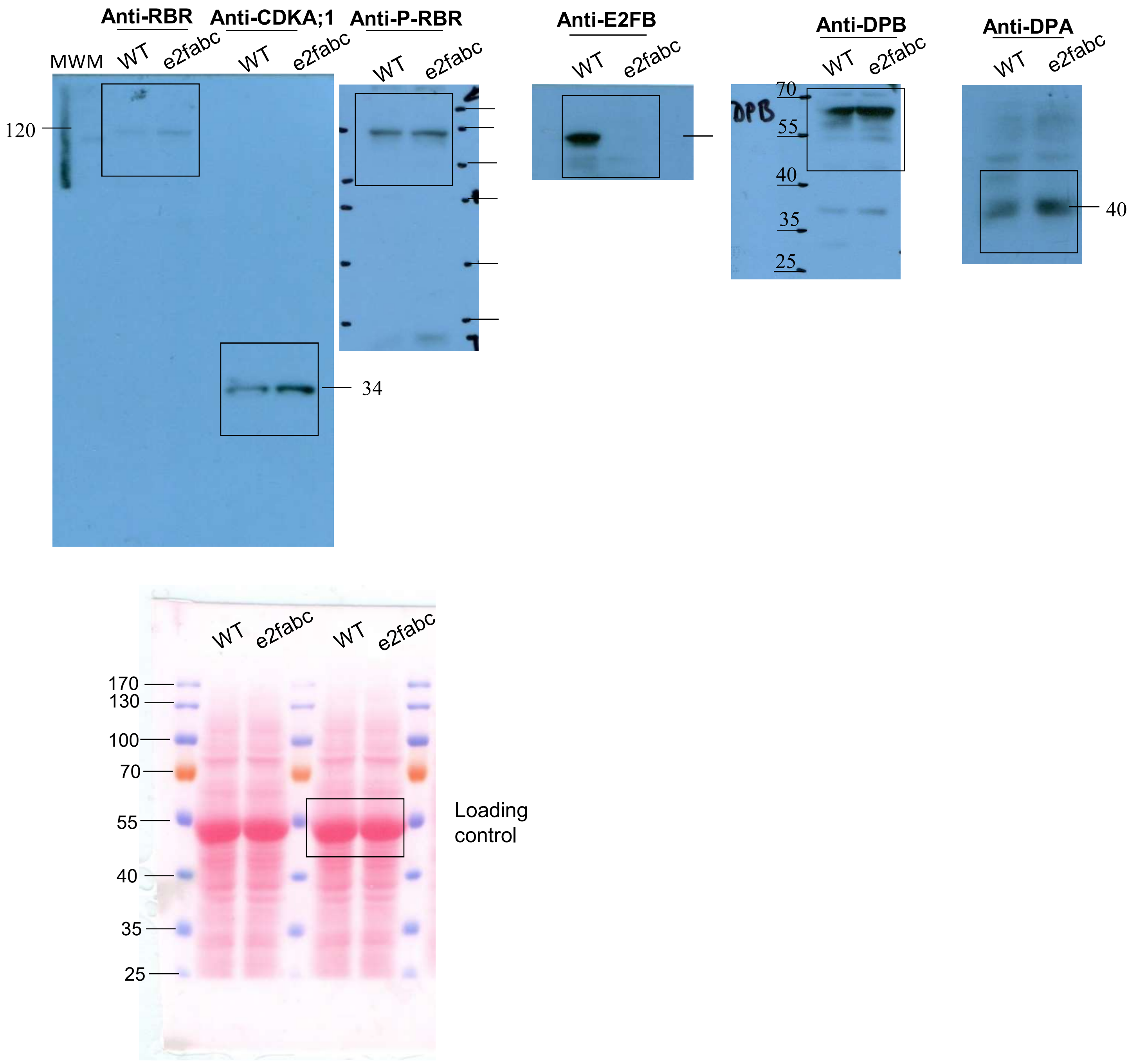

Supplementary Fig.4e and Fig.9b

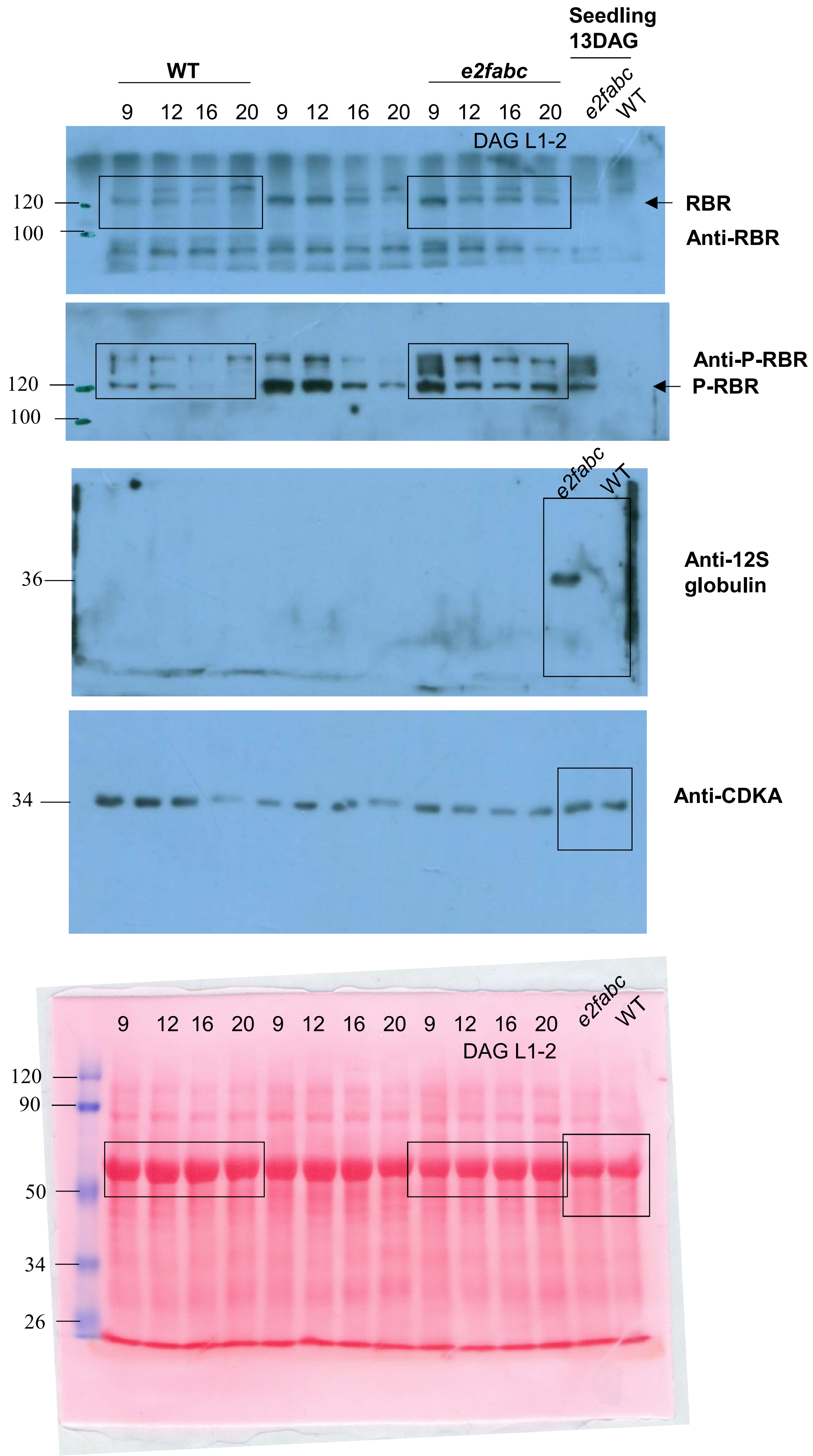

Supplementary Fig. 14. Raw, uncropped and unedited blot images of all western blots are shown in this study.
