# Supplementary material for: The canonical E2Fs together with RETINOBLASTOMA-RELATED are required to establish quiescence during plant development
Source: Commun Biol. 2023 Sep 4;6:903. doi: 10.1038/s42003-023-05259-2 (PMC10477330; doi:10.1038/s42003-023-05259-2)
Supplement: Supplementary file 3 — Description of Additional Supplementary Files [file 42003_2023_5259_MOESM3_ESM.pdf]

Description of Additional Supplementary Files

File name: Supplementary Data 1
Description: Differential expressed genes (DEG) in the *e2fabc* triple mutant plants compared to wild type plants.

File name: Supplementary Data 2
Description: Direct target genes bound by RBR, E2FA, E2FB, E2FC and MYB3R3 as defined by ChIP-seq analyses.

File name: Supplementary Data 3
Description: Target gene list of different E2F-MYB3R-RBR categories.

File name: Supplementary Data 4
Description: Source Data for the graphs are presented in this work.
