# Supplementary material for: The canonical E2Fs together with RETINOBLASTOMA-RELATED are required to establish quiescence during plant development
Source: Commun Biol. 2023 Sep 4;6:903. doi: 10.1038/s42003-023-05259-2 (PMC10477330; doi:10.1038/s42003-023-05259-2)
Supplement: Supplementary file 8 — Reporting Summary [file 42003_2023_5259_MOESM8_ESM.pdf]

nature portfolio

Corresponding author(s): Zoltán Magyar

Last updated by author(s): Aug 14, 2023

Reporting Summary

Nature Portfolio wishes to improve the reproducibility of the work that we publish. This form provides structure for consistency and transparency in reporting. For further information on Nature Portfolio policies, see our Editorial Policies and the Editorial Policy Checklist.

Statistics

For all statistical analyses, confirm that the following items are present in the figure legend, table legend, main text, or Methods section.

n/a	Confirmed	
☐	☒	The exact sample size (n) for each experimental group/condition, given as a discrete number and unit of measurement
☐	☒	A statement on whether measurements were taken from distinct samples or whether the same sample was measured repeatedly
☐	☒	The statistical test(s) used AND whether they are one- or two-sided<br>*Only common tests should be described solely by name; describe more complex techniques in the Methods section.*
☒	☐	A description of all covariates tested
☒	☐	A description of any assumptions or corrections, such as tests of normality and adjustment for multiple comparisons
☐	☒	A full description of the statistical parameters including central tendency (e.g. means) or other basic estimates (e.g. regression coefficient) AND variation (e.g. standard deviation) or associated estimates of uncertainty (e.g. confidence intervals)
☐	☒	For null hypothesis testing, the test statistic (e.g. F, t, r) with confidence intervals, effect sizes, degrees of freedom and P value noted<br>*Give P values as exact values whenever suitable.*
☒	☐	For Bayesian analysis, information on the choice of priors and Markov chain Monte Carlo settings
☒	☐	For hierarchical and complex designs, identification of the appropriate level for tests and full reporting of outcomes
☐	☒	Estimates of effect sizes (e.g. Cohen's d, Pearson's r), indicating how they were calculated

Our web collection on statistics for biologists contains articles on many of the points above.

Software and code

Policy information about availability of computer code

Data collection	No software was used for data collections
Data analysis	Confocal images for root, leaf and cotyledons were analyzed with Leica TCS SP5 confocal laser scanning microscope (Germany).<br>Organ and epidermal cell sizes were measured using ImageJ software.<br>For microscopic observations of cleared tissues with differential interference contrast (DIC) microscope (BX51, Olympus) images were captured with a CCD camera (DP74, Olympus) and an image capture software (CellSens Standard, Olympus).<br>qPCR results were analyzed using Microsoft Excell.

For manuscripts utilizing custom algorithms or software that are central to the research but not yet described in published literature, software must be made available to editors and reviewers. We strongly encourage code deposition in a community repository (e.g. GitHub). See the Nature Portfolio guidelines for submitting code & software for further information.

Data

Policy information about availability of data

All manuscripts must include a data availability statement. This statement should provide the following information, where applicable:

- Accession codes, unique identifiers, or web links for publicly available datasets
- A description of any restrictions on data availability
- For clinical datasets or third party data, please ensure that the statement adheres to our policy

NA

Research involving human participants, their data, or biological material

Policy information about studies with human participants or human data. See also policy information about sex, gender (identity/presentation), and sexual orientation and race, ethnicity and racism.

Reporting on sex and gender	NA
Reporting on race, ethnicity, or other socially relevant groupings	NA
Population characteristics	NA
Recruitment	NA
Ethics oversight	NA

Note that full information on the approval of the study protocol must also be provided in the manuscript.

Field-specific reporting

Please select the one below that is the best fit for your research. If you are not sure, read the appropriate sections before making your selection.

☒ Life sciences ☐ Behavioural & social sciences ☐ Ecological, evolutionary & environmental sciences

For a reference copy of the document with all sections, see nature.com/documents/nr-reporting-summary-flat.pdf

Life sciences study design

All studies must disclose on these points even when the disclosure is negative.

Sample size	Sample size was determined based on previous studies and experiments in the field. Sample size was indicated in the corresponding figure legends.
Data exclusions	No data was excluded.
Replication	All experiments were biologically repeated at least two times expect the ChIP-seq experiments. All attempts at replication were successful in this study.
Randomization	All samples were randomly collected.
Blinding	Blinding was not applied in our study. Experiment results are not subjective.

Reporting for specific materials, systems and methods

We require information from authors about some types of materials, experimental systems and methods used in many studies. Here, indicate whether each material, system or method listed is relevant to your study. If you are not sure if a list item applies to your research, read the appropriate section before selecting a response.

Materials & experimental systems

n/a	Involved in the study	
☐	☒	Antibodies
☒	☐	Eukaryotic cell lines
☒	☐	Palaeontology and archaeology
☒	☐	Animals and other organisms
☒	☐	Clinical data
☒	☐	Dual use research of concern
☐	☒	Plants

Methods

n/a	Involved in the study	
☐	☒	ChIP-seq
☐	☒	Flow cytometry
☒	☐	MRI-based neuroimaging

Antibodies

Antibodies used

Chicken anti-RBR antibody (Agrisera), mouse monoclonal anti-PSTAIRE (CDKA;1 specific; Sigma), anti-phospho-specific Rb (Ser-807/811) rabbit polyclonal antibody (Cell Signaling Tech), anti-DPA, anti-E2FB polyclonal rabbit antibodies (Magyar et al., 2005), anti-DPB polyclonal rabbit antibody (Umbrasaite et al., 2010), and anti-E2FA rat polyclonal antibody (Leviczky et al., 2019), rabbit polyclonal antibody anti-12S globulin (Shimada et al., 2003), and anti-GFP antibody (Abcam, ab290).

Validation

All antibodies used in this study were experimentally tested and validated; anti-RBR (used in 1:2000; Agrisera), anti-PSTAIRE (used in 1:40000; Sigma), anti-phospho-specific Rb (Ser-807/811 - 1:500; Cell Signaling Tech; successfully used in plants, eg Magyar et al., 2012 EMBO J 31: 1480-93; Wang et al., 2014 Cell Host Microbe 16: 787-94), anti-DPA (1:400), anti-E2FB (1:400) and anti-DPB (1:400) antibodies (Magyar et al., 2005 Plant Cell 17: 2527-41; Umbrasaite et al., 2010 PLoS One 5: e15357), anti-E2FA (1:400; Leviczky et al., 2019 Development 146), anti-12S globulin (1:10000; Shimada et al., 2003), anti-GFP antibody (Abcam; this antibody has been used successfully for several ChIP-seq analyses in our lab and is highly rated by multiple customers for ChIP https://www.abcam.com/content/anti-gfp

Dual use research of concern

Policy information about dual use research of concern

Hazards

Could the accidental, deliberate or reckless misuse of agents or technologies generated in the work, or the application of information presented in the manuscript, pose a threat to:

No	Yes	
☒	☐	Public health
☒	☐	National security
☒	☐	Crops and/or livestock
☒	☐	Ecosystems
☒	☐	Any other significant area

Experiments of concern

Does the work involve any of these experiments of concern:

No	Yes	
☒	☐	Demonstrate how to render a vaccine ineffective
☒	☐	Confer resistance to therapeutically useful antibiotics or antiviral agents
☒	☐	Enhance the virulence of a pathogen or render a nonpathogen virulent
☒	☐	Increase transmissibility of a pathogen
☒	☐	Alter the host range of a pathogen
☒	☐	Enable evasion of diagnostic/detection modalities
☒	☐	Enable the weaponization of a biological agent or toxin
☒	☐	Any other potentially harmful combination of experiments and agents

Plants

Seed stocks

Arabidopsis thaliana Col-0 ecotype was the WT and background of all transgenic lines used in this study. T-DNA insertion lines and their crosses were used in this work: e2fb-1 (SALK_103138), e2fa-2 (GABI-348E09), e2fc-1, (GK-718E12), e2fc-2 (SAIL-1216G10);

e2fa-2/e2fb-1; e2fa-2/e2fc-1; e2fb-1/e2fc-1; e2fa-2/e2fb-1/e2fc-1; e2fa-2/e2fb-1/e2fc-2. Transgenic lines expressing E2FA-GFP, E2FB-GFP, E2FC-GFP, RBR-GFP, RBR-3xCFP, RBR-RFP and CYCB1;2-YFP have been previously generated.

Novel plant genotypes

E2fabc-2 triple mutant was generated by crossing the e2fab double (e2fa-2/e2fb-1) with the e2fc-2. By crossing, we also generated an e2fabc triple mutant line expressing the CYCB1;2-YFP marker.

Authentication

E2FA-GFP and RBR-GFP (Magyar et al., EMBO J 31, 1480-1493. 2012)
E2FB-GFP (Őszi et al., Plant Physiol 182, 518-533, 2020)
E2FC-GFP (Kallai et al., J Exp Bot 71, 1265-1277, 2020)
RBR-3xCFP (Leviczky et al., Development 146, 2019)
RBR-RFP (Biedermenn et al., EMBO J 36, 1279-1297, 2017)
CYCB1;2-YFP (Iwata et al., Plant Cell 23, 4382-4393, 2011)
e2fa-2/e2fb-1 (Heyman et al., Plant Cell 23, 4394-4410, 2011)
e2fa-2/e2fb-1/e2fc-1 (Wang et al., Cell Host Microbe 16, 787-794, 2014)

ChIP-seq

Data deposition

☒ Confirm that both raw and final processed data have been deposited in a public database such as GEO.

☐ Confirm that you have deposited or provided access to graph files (e.g. BED files) for the called peaks.

Data access links
May remain private before publication.

https://www.ncbi.nlm.nih.gov/geo/query/acc.cgi?acc=GSE218481

Files in database submission

GSM6745996_E2FA-GFP_q005_peaks.broadPeak.gz
GSM6745996_E2FA-GFP_S7.S3norm.bigwig
GSM6745997_E2FB-GFP_q005_peaks.broadPeak.gz
GSM6745997_E2FB-GFP_S8.S3norm.bigwig
GSM6745998_E2FC-GFP_q005_peaks.broadPeak.gz
GSM6745998_E2FC-GFP_S5.s3norm.bigwig
GSM6745999_RBR-GFP_q005_peaks.broadPeak.gz
GSM6745999_RBR-GFP_S6.s3norm.bigwig

Genome browser session
(e.g. UCSC)

NA

Methodology

Replicates

Each ChIP-seq experiment was done with two biological replicates

Sequencing depth

E2FA: total num 59522379; uniquely mapped reads: 35787887; length of reads: 76bp; single-end.
E2FB: total num 62993350; uniquely mapped reads: 40743647; length of reads: 76bp; single-end.
E2FC: total num 64666726; uniquely mapped reads: 36104297; length of reads: 76bp; single-end.
RBR: total num 81607431; uniquely mapped reads: 62551637; length of reads: 76bp; single-end.

Antibodies

anti-GFP antibody (Abcam, ab290)

Peak calling parameters

For read mapping:
bowtie2 -x bowtie_index_file -p 8 --very-sensitive -U clear_data.gz | samtools view -bS - > output.bam
samtools view -h -b -q 30 input.bam > aboveMAPQ30.bam
For peak calling:
macs2 callpeak -t sorted_dedupPCR_aboveMAPQ30.bam -g 119481543 -p 0.05 --nomodel --extsize 150 --bw 500 -B --outdir output

Data quality

Data quality control: use Trimmomatic remove adapter (ILLUMINACLIP:TruSeq3-SE.fa:2:30:10), remove leading low quality or N bases (below quality 5), remove trailing low quality or N bases (below quality 5) and drop the read if it is below 30bp, check mapping rate for each sample and remove the low quality reads (MAPQ < 30).
E2FA: FDR %5 27771; above 5 -fold enrichment: 0.
E2FB: FDR %5 26356; above 5 -fold enrichment: 0.
E2FC: FDR %5 24883; above 5-fold enrichment: 0.
RBR: FDR %5 27089; above 5-fold enrichment: 0.

Software

Trimmomatic-0.38: performs trimming tasks for ChIP-seq data.
bowtie2/2.3.5: a tool for aligning ChIP-seq sequencing reads to reference genome.
Samtools 1.16.1: processing ChIP-seq Sequence Alignment/Map (SAM) format files and generate index files.
Bedtools 2/2.28.0: convert bam file to bed file of ChIP-seq.
Deeptools 3.5.0: takes an alignment of reads as input (BAM file) and generates a coverage track (bigWig) as output.
macs2 2.2.7.1 : peak calling for ChIP-sequencing experiment.

Flow Cytometry

Plots

Confirm that:

- [ ] The axis labels state the marker and fluorochrome used (e.g. CD4-FITC).
- [ ] The axis scales are clearly visible. Include numbers along axes only for bottom left plot of group (a 'group' is an analysis of identical markers).
- [ ] All plots are contour plots with outliers or pseudocolor plots.
- [x] A numerical value for number of cells or percentage (with statistics) is provided.

Methodology

Sample preparation	The first leaf pair and the cotyledons were collected and chopped by razor blades in nuclei extraction buffer and stained with DAPI.
Instrument	Partec PAS2 Particle Analysing system (Partec, Germany)
Software	Data were analyzed with Flomax software (Partec).
Cell population abundance	No cell sorting was done. The data represent one parameter flow cytometry measurement of DAPI stained DNA content. All peaks satisfied the criteria showing double mean values. Peak data distribution was within 10% CV
Gating strategy	No gating has been used. The DAPI-stained DNA distribution was analysed by the Partec peak analysis software package.

- [ ] Tick this box to confirm that a figure exemplifying the gating strategy is provided in the Supplementary Information.
